# Understanding the ups and downs of living well: the voices of people experiencing early mental health recovery

**DOI:** 10.1186/s12888-018-1703-1

**Published:** 2018-05-04

**Authors:** Nicola Hancock, Jennifer Smith-Merry, Glenda Jessup, Sarah Wayland, Allison Kokany

**Affiliations:** 1 The University of Sydney, Sydney, Australia; 2 University of Technology, Sydney, Sydney, Australia; 3 WentWest, Blacktown, Australia

**Keywords:** Early recovery, Recovery, Mental illness, Experience, Severe and complex mental illness

## Abstract

**Background:**

The aim of this study was to better understand early-stage mental health recovery experiences of people living with severe and persistent mental illness and complex needs.

**Methods:**

Semi-structured, in-depth interviews were conducted with 13 people engaged in an Australian program specifically designed for people facing complex barriers to their recovery. Interview data were analysed thematically using constant comparative methods.

**Results:**

Participants described engaging with seven interconnecting aspects of early recovery: (1) engaging with the challenge of recovery; (2) struggling for a secure and stable footing; (3) grieving for what was and what could have been; (4) seeking and finding hope; (5) navigating complex relationships; (6) connecting with formal and informal support, and finally, (7) juggling a complexity of health issues.

**Conclusions:**

This study illuminated the complexity of earlier-stage recovery which was characterised both by challenging personal circumstances and a hope for the future. It illustrated that even at an early point in their recovery journey, and amidst these challenging circumstances, people still actively engage with support, draw on inner strengths, source resources and find accomplishments. Stability and security was foundational to the ability of participants to draw on their own strengths and move forward. Stability came when material needs, including housing, were addressed, and an individual was able to connect with a supportive network of workers, carers, friends and family.

## Background

Consistent evidence now refutes previously pessimistic views (e.g., [1, 2]), that severe and persistent mental illnesses such as schizophrenia, are inevitably degenerative and life-long. A multitude of large, longitudinal studies evidence that at least a quarter of people living with severe and persistent mental illnesses such as schizophrenia, will recover in a curative or clinical sense [3]. However, rather than this curative framing or understanding of recovery, mental health systems and policies internationally have adopted a different, personal or consumer-defined, understanding of recovery. This personal recovery is witnessed in published testimonies of consumers or people living with mental illness themselves as well as the analysis and synthesis of first-person qualitative data [4, 5]. These accounts emphasise an individualised, ongoing and non-linear journey towards living a life of personal meaning and value irrespective of whether symptoms of mental illness persist or not (e.g., [6, 7]). This understanding of recovery, is the focus of the current study.

While the language of personal recovery is now visible across mental health policies, system guidelines and plans internationally (e.g., [8–10]), there remain challenges regarding the utility and relevance of dominant discourses and understandings of recovery across the diversity of experiences for those living with mental illness.

Critics have also challenged conceptualisations and models developed to operationalise the construct of recovery as too optimistic. The literature lacks a focus upon the more complex process of recovery experiences by many living with severe and persistent mental illness at earlier points in their recovery journey [11]. It is therefore argued that, without a more inclusive conceptualisation or model, recovery is not accessible or considered relevant or relatable to many individuals commencing their recovery journey, or to those supporting them [12, 13].

There has been a call for a more nuanced understanding of recovery which, while focusing on the positive parts of the recovery journey, also acknowledges and respects the perhaps more challenging earlier recovery experiences. This more inclusive conceptualisation would place more emphasis on difficulties and complexity as a valid and normal part of recovery. Authors [14], in a meta-synthesis of 12 qualitative studies of recovery, evidenced that there are indeed complexities and difficulties that are not encapsulated in dominant recovery discourse and models. Comparing the data against the much heralded CHIME (Connectedness, Hope and optimism, Identity, Meaning, Empowerment) recovery model [15], they found that only 70% of data fitted within the relatively optimistic themes of this model. The authors coded the remaining data under four additional themes, one of which they labelled “Difficulties”, encompassing: ambivalence and contradiction; disempowerment; financial concerns; loss and negative life changes; stumbling, struggling and suffering and substance use comorbid with mental illness. Difficulties was the second most frequently occurring theme overall, second only to the CHIME theme of Empowerment. They argued for a conceptualisation of recovery in which difficulties and struggles are more prominently considered, so that an overly optimistic view of recovery does not result in professionals’ homogenising or blaming individuals [14].

While recovery is understood as an individual and often non-linear journey, numerous authors have proposed that a person’s recovery process involves a process of early, mid and later stages. There has been less focus upon recovery’s early stages and the literature on stages may thus be less relevant or meaningful to those at this earlier point on their recovery journey. This is perhaps because a staged understanding of recovery is typically informed by those at later stages of recovery reflecting back on their experiences, rather than being informed by in-the moment experiences [14, 16]. It might also be because of a desire, in line with recovery philosophy, to focus upon the positive. For these reasons, and likely others, we have a much poorer understanding of the early-stage recovery experiences and achievements than we do of later stages [14, 17].

With these considerations in mind we sought to understand the early recovery experiences of people engaged in a Sydney-based Partners in Recovery (PIR) program. This national, government-funded, program was established to support individuals with severe and persistent mental illness and complex needs or barriers (e.g. homelessness, drug-use, physical ill-health) to engaging in their recovery. Central to PIR programs are the support facilitators who meets with individuals to understand their needs and goals and then seek out and bring together the services needed. The PIR initiative, including the demographic profile of program participants, their needs and the role of the Support Facilitator is described in further detail elsewhere [18–20]. While the program, as indicated in its name, has a specific orientation to recovery, there was little understanding of who this group were and their specific recovery needs when it was initiated [21]. There was also little literature that spoke directly to this group which could be drawn on to either assist support facilitators in their work or to provide PIR clients with a realistic representation of what recovery may involve for them. For that reason we set out to better understand the recovery experiences of people assessed as currently experiencing a complexity of needs and multiple barriers to engaging in their recovery.

## Methods

### Aims and study design

This project aimed to better understand the recovery experiences of people with severe and complex mental illness. A qualitative approach built around semi-structured, in-depth interviews was adopted in order to develop a rich understanding of participants’ experiences of mental health recovery. Pseudonyms are used to protect participant confidentiality. The study practices were aligned with the consolidated criteria for reporting qualitative research (COREQ) [22].

### Participant recruitment

Participants were recruited from a Sydney-based PIR program operating in an area of high social disadvantage. The demographic profile of PIR participants in this program has been reported elsewhere [23]. To be eligible for the study, potential participants needed to be deemed: 1) able to provide informed consent, and 2) able to participate in an interview without the interview being likely to cause significant distress. This was determined by Support Facilitators who identified eligible potential participants. These individuals were sent an invitation to participate by the PIR administration staff. A participant information sheet containing the interview questions was included with the invitation letter so that potential participants understood what would be covered in the interviews. Consumers were asked to contact the research team directly if they wanted to participate in the research. Participants received a $20AUS grocery card as appreciation for their time and stories.

### Data collection

Semi-structured, in-depth interviews were facilitated by the use of an interview guide. The guide consisted of broad questions focused around the topic of inquiry such as “What has helped or made you feel good as part of your recovery journey?” and “What has caused difficulties for you on recovery journey?” The guide was designed to ensure all pertinent areas were explored, while providing enough flexibility to ensure participants could elaborate on aspects of recovery that were most pertinent to them.

Each participant’s Support Facilitator was present at the interview location (although not in the room unless requested). During the interview, the trained interviewer (SW) checked for participant distress by monitoring each participant’s responses and non-verbal cues. If participants became distressed they were asked if they wished to take a break or to stop the interview. Participants were given an opportunity to speak with their support facilitators after the interview to ensure they were not unduly distressed as a result of the interview. Interviews took between 22 and 74 min and were recorded, transcribed verbatim, and summaries sent to each participant for the opportunity to remove any materials that they did not want included in the final study. No data was requested to be removed.

### Data analysis

Data were entered into NVivo 10 [24] and analysed using constant comparative analysis [25, 26]. The first stage of analysis involved a process of line-by-line coding to label small segments of data to summarise concepts [26]. A code could be a word, sentence, or paragraph that expressed a single idea, feeling, experience or topic. As this analysis continued, data and codes were constantly compared within and between transcripts. Two authors (GJ and NH) independently coded the first two transcripts, and then came together to discuss, compare, and reach consensus on early coding decisions. Subsequent transcripts were coded by author GJ. The next stage of analysis, focused coding, involved examining the relationship between codes and synthesising or drawing together those that were conceptually overlapping into broader conceptual categories [26]. These categories also included examples of exceptions, contradictions and contrasts. Throughout the analysis, reflexive discussions were conducted between authors (GJ and NH) to ensure emerging codes were representative of the data, enhancing coding rigor [26]. A third author independently reviewed all coding (JSM).

## Results

### Participants

Thirteen consumers, eleven female and two males, contacted the research team and were interviewed. In keeping with the PIR inclusion guidelines all had a severe and persistent mental illness with a complicating co-morbidity, including physical ill-health or addiction. To mimimise risk of distress, participants were not directly asked about their diagnoses, however through interview data, it was evident that: most had more than one psychiatric diagnosis; five were currently grappling with additional drug and or alcohol addiction, and almost all described a range of physical health co-morbidities, including three who were concurrently grappling with cancer. Six participants were currently in precarious accommodation. All participants were aged between 18 and 65, the criteria for PIR registration. Nine participants described lengthy periods as an inpatient within a psychiatric hospital, with one person having recently spent over 30 years in an inpatient facility (Table 1).

All participants described their experience of mental health recovery as an ongoing, active, challenging, but ultimately positive journey of ups and downs. Our analysis of the data brought to light seven inter-connected, over-arching experiences that patients spoke about as central to their recovery journeys including: (1) engaging with the challenge of recovery; (2) struggling to find safety and security; (3) grieving for what was and what could have been; (4) seeking and finding hope and purpose; (5) navigating complex relationships; (6) connecting with formal and informal support; and finally, (7) juggling a complexity of health issues. These are detailed below. Quotations are an important part of reporting qualitative research. They provide illustration and evidence that findings and interpretations have arisen from the data [22, 27]. We utilise quotations extensively to demonstrate as much as possible what recovery meant for participants in their own words.

**Table 1:** Early mental health recovery: themes and sub-themes

1. Engaging with the challenge of recovery	
*a* it needs to be me	
*b standing up for myself*	
*c fighting the desire to withdraw or give up*	
*d accepting support and that recovery can’t be ‘done’ alone*	
*e struggling with stigma and learning to be king to yourself*	
2. Struggling to find safety and security	
3. Grieving for what was and what could have been	
4. Seeking and finding hope and purpose	
5. Navigating complex relationships	
*a* managing the impact of illness on others	
*b* separating carer roles from family and friends	
*c* managing and avoiding unhelpful relationships	
6. Connecting with formal and informal support	
7. Juggling a complexity of health issues	

#### Engaging with the challenge of recovery

Repeatedly participants used the word ‘fight’ to describe the internal processes involved in their recovery. This internal fight involved: coming to terms that it needs to be me; standing up for myself; fighting the desire to withdraw or give-up; accepting support and that recovery can’t be done alone; and struggling against stigma – learning to be kind to yourself.

##### It needs to be me

All participants talked about coming to the realisation that they needed to be active in their own recovery. Lara reflected on this process: “*I believed I could go to a doctor… I believed there was a magic pill. The only magic pill is from within…. you have to make that step, and it’s scary.”* Some participants were very clear and resolute, for example, Naree said: *“I have to do this for myself. Nobody can do it for me”*. Suzy commented, “*I’m not going to be able to progress….if I don’t find a way to deal with… those issues that I now know I have.”* Other participants grappled with this, and their sense of agency fluctuated: “*I feel like sometimes I’m trying to climb up a hill and I just keep getting pushed back down, so I just sit there and think, well, bugger it. But then I find after a day or two… I get back [up]… I get this inner strength*” (Lara).

##### Standing up for myself

Participants repeatedly discussed the difficulty but importance of standing their ground, challenging advice and instructions, and standing up against clinical views and practices that they did not agree with. They talked about the fight to get the right medication and treatment. Most participants felt that they were often not listened to when it came to medication. Ann said, “*They were medicating me, but not listening too… it was horrible.”* Similarly, Suzy said *“within 20 minutes [psychiatrist] had given me all these diagnoses, which are true, but the medication - he put me on medication with 20 minutes of meeting me, not even knowing too much about me.”* Ryan commented, *“GPs and all that … after a couple of sessions they just want to prescribe you medication.”* Ann challenged the clinical treatment she was receiving: *“…before I saw my current psychiatrist I saw another one. If I said to her I feel a bit anxious [her response was] “have another pill”, to the point where I asked in the end for a second opinion whilst I was an inpatient, and ended up seeing my current psychiatrist for a second opinion. He flipped. He* [said] *you are on double the recommended doses of some of this stuff.”* Standing up to professionals however, particularly when it came to medication or being believed, was not always easy. As Ann commented, *“It’s hard to question professional advice.”* At this early stage on their recovery journey participants did not always feel able to stand up for themselves and argue for their needs. Questioning was made easier if participants felt validated by other professionals. Coralyn’s counsellor, for example, encouraged her to question treatment decisions, by saying to her *“… stand for your right. When you don’t have the answer, when you don’t ask… how are you going to find your answer?”*

##### Fighting the desire to withdraw or give-up

Another internal fight repeatedly described by participants was the battle against a desire to give up or withdraw from the process of recovery. Participants talked about fighting against internal negative impulses, thoughts, poor coping habits, or self-doubt. Ann’s *“constant fight”* was against negative thoughts, *“they drive me batty”*, while Suzy fought the desire to give up on life, *“At the very last moment before I try to commit suicide I’d be saying… ‘What are you doing? Don’t stop fighting, don’t give up.’”* Julie fought the impulse to isolate herself from others, *“I’m trying to get my life where I shouldn’t have to keep myself in my house.”* Suzy and Naree spoke about fighting against and sometimes giving in to drugs and alcohol which they had adopted as coping habits.

##### Accepting support and that recovery can’t be ‘done’ alone

Participants described coming to a point of realising, they could not, and did not have to, strive for recovery alone. They described a sometimes challenging process of accepting that they needed help, formal or informal, from others. Dana reflected, *“I do strive for independence, but at the same time, I’m aware that I need a network of support…to be independent.”* Lara echoed Dana’s sentiment, *“… you need support. Without support, it can’t be done.”* However, learning to ask for and accept help did not always come easily. Dana, found it *“quite hard.”* She commented, “*Three years ago, I could have got all this help… but I don’t know if I would have been strong enough to accept all that help at the same time.”* Kerry also had difficulty asking for help: *“I’ve been a proud independent person and I’ve earned good money over the years.... I hate having to ask for help.”*

##### Struggling with stigma and learning to be king to yourself

Participants described struggling against their own and others’ judgements and stigma, and the need to learn to be kind to themselves. They repeatedly spoke about encountering stigma from people in their communities. Participants recounted how both their own and others’ stigma surrounding mental illness affected their preparedness to disclose, and their relationships with others. Suzy commented, “I *need people to know,… for my growth and my benefit,… I need them to know about mental health. It’s not scary and it’s not wrong*.” Dana, who initially did not disclose or discuss her mental illness with her family, “*we didn’t talk about it very much at all. Yeah, so it pretty much wasn’t there”* talked about the value of them ultimately understanding and *“not... pretending… you* [the family] *know”.* Kerry could not disclose to her church friends, *“Mental health within church circles isn’t always widely accepted as a ‘thing’. They can deal with a broken leg or a broken back but mental illness they don’t deal well with.”* Another participant (Ann) was cut off from family contact because of her mental illness. Stigma from others compounded the self-judgement participants levelled at themselves. Lara said that she had become, *“antisocial, to protect everyone else from me.”* Some participants talked about finding the strength to overcome self- and others’ judgement and about starting to be gentler and kinder to themselves. Kerry, for example, reflected on the importance of, *“learning to be kinder to myself on those days when I’m not having a good day.”* Yvonne, described her strength in overcoming previously hurtful comments from others: *“you’ve got a choice either to let it get to you or... use it to… gain your inner strength.”*

#### Struggling to find safety and security

All but one participant described struggling for immediate safety and security in their lives. This included the need for physical safety, secure and stable housing, food and basic daily necessities, and reliable, dependable support services. Malcolm, for example, wanted reassurance, *“that my future is safe.”* Naree needed a safe place to live stating that “*stability is the home…. There’s a roof. It’s very important... Once I have stability I’m fine”* Brenda commented, that *“people don’t realise how much [difficult] financial states of things… can add to your mental health and the strain that it puts on you.”* Housing stability was emphasised most frequently with lack of money exacerbated participants’ housing instability. They described being subject to short rental periods, having nowhere to go when their lease expired, lived in substandard housing, or in neighbourhoods in which they felt unsafe. Eileen for example felt *“terrified”* and unsafe in her public housing, ***“****You can’t walk the streets…you’re living around ice addicts and they’re always breaking into my backyard…. I get scared for my son.”* Participants escaping domestic violence had spent time in refuges, often with their children. Others had spent time being homeless. Yvonne, the only participant who did not express a need for stability, lived in a *“tight-knit family”* and drew on them for stable support.

Desperate attempts to meet these basic needs sometimes led to participants making decisions, actions and choices they later regretted. Sometimes, to fulfil their basic needs, participants put themselves at risk physically or legally. For example, Suzy, *“not thinking very clearly whatsoever, just the desperation of wanting to get somewhere”* agreed to a stranger visiting her home to discuss accommodation. *“He arrived at my place and he sexually assaulted me…. I should have thought about it better.”* Naree, who, with her young daughter, had escaped domestic violence, recounted, *“I was falling behind in my rent…. before I know it I’m in mess and it’s really hard to catch up.”* She tried to sell drugs to make some quick money and was arrested, *“life slapped me so hard…. I thought, that’s it, I’m screwed up and now …I’ve definitely lost [daughter].”*

The stability of affordable support services was also regarded as a basic need. Lara reflected on unreliable services and *“constantly… getting let down”* with, *“counsellors saying, ‘all right, your time is up now’, your file goes there and you’ll be okay’.”* She contrasted these experiences with her current counsellor who said*, “You belong here whenever you want to belong here.”* Stability of support services could be compromised by instability in the individual’s own lives. Naree’s frequent accommodation changes, for example, impacted on the continuation of her support services: *“every time I had someone calling… to catch up and see me, I was changing location.”* She continued, *“By the time they get their arse there I’m [at] a different address. So they go, ‘okay, that’s out of my area’…. They were not interested in me… Even though I was screaming out for help.”* Financial barriers also reduced service stability. Lara said, “*the money gets too high, the cost.”*

#### Grieving for what was and what could have been

Participants described grieving over the losses they experienced due to mental illness. These included: loss of a previous identity, loss of dreams and aspirations, and loss of family and social networks. Kerry said, *“I didn’t realise how much…my identity was tied up into being a nurse.”* Ann “*cried buckets over the fact that I can’t work…I’m no longer the same as everybody else.*” Dana lost her job and with it, her social network, “*when I took the work away…there was just me at home with the kids…. there are no networks there.*” Suzy also struggles to find her purpose in life, *“I’m trying to find out who I am and what my purpose is here.”* Her dream, “*was to have a husband and kids and that’s what I’ve wanted since I was little*.” Instead she felt that she had lost *“everything*” because of her heroin use. Ryan’s aspirations changed when he went to jail, “*I had everything, I was working, everything.”* Coralyn, who took great pride in her appearance, gained weight on her medication. She struggles to reconcile with this new self-image, *“I’m short and the tiny bones, and just getting fatter and fat…and lots of puffiness.*”

#### Seeking and finding hope and purpose

Alongside living with loss, participants described seeking and finding purpose and hope for their future. Lara’s recovery defied others’ messages of hopelessness: *“I used to think that I wasn’t worth helping…that they’d come to the end of the road with me…. I have been told by people that nothing will ever change”* Hopelessness was, *“horrible… I’d just go home and cut myself.”* Over time Lara came to new realisations, *“But now I realise, of course there’s hope.”*

Hope supported people to seek and finding purpose and meaning through doing positive things both for themselves and for others. Life was more meaningful when participants were doing positive things for themselves: “*you have to put yourself first.”* (Julie). Yvonne loved long nature walks, *“I go for walks, I sing out loud.”* Malcolm wrote poetry, *“My poems [are] something special…. I write unique stuff…. I always remember myself as an inheritor of my own contribution to life on earth.”* Participants also valued structure in their day. Ann, for example, needed *“something on each day to try and keep me well and accountable.”*

Beyond doing things for themselves, participants found affirmation and purpose in doing things for others. Ann gave her artwork to friends. Suzy helped her neighbour, *“they’re struggling. I’ve got some spare bed frames and stuff, she needs a bed and they need food vouchers. So I ran around and got all the numbers and made appointments for stuff like that.”* Kerry is training as a counsellor. She feels her experiences have qualified her to help others, *“I went through all of that for the here and now… for me to help others… you can’t look at it any other way.”*

Seven participants were parents. This parenting role gave them a sense of being needed by another even when their children were now adults. Supporting, protecting, guiding and seeking to re-gain custody of children was a central life purpose for most participants. Parents described finding purpose and accomplishment in meeting their children’s needs and routines. Julie was doing new things for her children which she felt proud of, *“changing eating habits…. Setting them some little goals…. That’s new for them.”* Naree reflected, *“I came out better because [daughter’s] there…You realise how beautiful that little thing has been in your life … still hugs you and says, ‘Mummy I love you.’”* They also found purpose in being good role models for their children, or giving them a better life than they had experienced. Lara commented, *“Everything I do is for [son], because I don’t want him to see me as a failure. I want him to understand that… you’ve got to take a step forward to get somewhere.”* Eileen’s energies were directed towards her youngest son’s future, *“I fought too hard to keep this boy alive. I’m not going to sit back and watch him go down the drain and become a street buck. He’s better than that.”*

For some participants, religious faith provided hope and purpose in life. Participants however, described needing to reconcile why they had been subjected to deep suffering. Ann found hope in her belief that *“Jesus will come again and there will be no more sadness, no more tears, no more pain, no more suffering.”* In contrast, Suzy said *“there’s a bit of faith left… I’ve got some renewed faith”* but*“I’m still finding it hard to believe that there’s something out there that wants good things for me, because I’ve got nothing but pain.”* She described being *“envious”* of people who had a strong faith, *“I want something like that.”*

#### Navigating complex relationships

Participants spoke about navigating complex friend and family relationships. They described navigating: a) the impact of their illness on others; b) the need to separate carer roles from friends and family, and c) managing and avoiding unhelpful relationships.

##### Managing the impact of illness on others

Participants expressed concern about the impact their illness might have on friends and family. Suzy for example, felt that her good friend should not have to be *“putting up with my crap.”* Lara, described being *“very upfront”* with her young son about her illness, but striving to provide him with age-appropriate explanations for what he witnessed, *“I had to, otherwise he would have been a very screwed up kid.”*

##### Separating carer roles from family and friends

Participants also struggled to keep carer roles separate from friend and family relationships. Ann’s friends, *“were starting to take over”* as carers. This became a source of tension, “*I like to keep my friends as friends and my carers as carers.”* Equally, the adult children of some participants assumed carer roles and participants felt ambivalent about this. While acknowledging its necessity, some described a sadness about this blurring of roles. Julie for example reflected, “*I don’t like … that, because I’m the parent. I’m the one that’s supposed to be supporting people, not them supporting me… I shouldn’t have to speak to her [daughter] because I’m a parent.”* and Lara recounted “*My son had to do everything for me, take me shopping, talk for me, ring up people.”* Ann’s support services helped her negotiate the tension of friends becoming carers by sourcing personal carers for her.

##### Managing and avoiding unhelpful relationships

For most participants, family relationships were exceedingly complex to negotiate. Family members could support or hinder recovery. Eileen, who had isolated herself from friends who were drug users to live *“a very lonely”* but *“safe”* life, found it more difficult to isolate herself from her brother, a drug user, with whom she shared accommodation, *“I was so angry and disappointed that my brother was using drugs under my roof.”* Ann was one of a number of participants whose family had stopped having contact with her. She replaced this loss with her *“church family”*, but still found traditional festive times difficult: *“They’re* [biological family] *all together for birthdays, Christmas, Easter…I’m just not welcome there.”* Coralyn needed protection from an emotionally abusive husband, whom she could not afford, financially, to leave. She battled to have her reports of abuse believed and not dismissed as “*a mental person making a story”*. Being vindicated, helped her endure, “*I have someone to talk and believe, and help….my psychologist believes me, my friends believing me… People who can support me and stand behind… me.”* Ann and Naree described needing to set boundaries in their relationships with their mothers. Ann commented, *“Probably the best thing that I’ve done in terms of my wellness journey is really limiting the amount of time I’m with my mother…she’s nasty when she drinks.”* Naree’s *“relapse prevention strategies”* included not depending on her mother because: *“You [mother] just take off…. By the time you come back, I’ve already done the grieving …. picked myself up …going stable... then you turn up.”*

#### Connecting with formal and informal support

Participants described two important connections that supported their recovery. These were support workers and informal supports that included peers, helpful friends and family members. Participants highly valued the help they received from their Support Facilitators and other mental health workers or professionals. They valued their: persistence; care in checking on them through the ups and downs; accessibility; ability to facilitate access to resources; belief in them, and their focus on them as individuals. Julie said, *“…ever since I’ve met [Support Worker]… she’s actually made me a little bit stronger... I know that sounds stupid, meeting somebody that you’ve only known for a few months who’s given you the courage and the knowledge to be able to go places.”* Kerry, explaining the difference between friends and her Support Worker, stated *“I had my friends, but they didn’t understand. They just knew that I was doing things that weren’t safe…. If it hadn’t been for [Support Worker] walking beside me, just being that strength there for me I wouldn’t have got through.”*

The personal connection and accessibility of workers was important to participants. Ryan, for example commented, “…*if I get too worried, I’ll talk to [support worker], I’ll ring her up for support.”* Julie said, “*You feel like you can speak to her about anything”.* Naree described feeling valued, *“…the fact that she shouted me coffee and she said, do you want something to eat? It was great because you feel like, wow, you feel important. I haven’t felt like that for a long time.”* Ann said her psychiatrist, *“…respects me, and he has said to me on numerous occasions ‘You’re the expert in this, you tell me’, and that’s good.”*

However participants did not feel this connection with all mental health workers. *“I’ve got that connection to [Support worker] that I can’t seem to get with a psychologist. I think it’s because I trust her and she knows a lot about what’s going on. She’s seen me in my ups and she’s seen me in my downs, where they haven’t.”* The support workers that participants described as ‘successful’ at engaging or connecting were those that were personable, valued them as individuals and were able to connect them with services and supports that facilitated their recovery, such as housing (Kerry; Julie), the Police (Suzy), financial advisors (Dana), the National Disability Insurance Scheme and physical health support (Brenda). Participants spoke of the importance of working in a team with support workers to identify and source services. Brenda enjoyed feeling part of a team when exploring support options and resources, “[*support worker*] *said,” “I don’t know a lot about it” and I said, “neither do I.” “So we went as like a team together.”*

Some participants also described the value of connecting with peers, or others with their own lived experience of mental illness. Lara for example, felt *“inspired”* by peer workshops, *“I take everything I can with me and use it in day to day life…you do go home knowing that you’re not alone and you can make friends.”* Peer connection was important to Lara as she had isolated herself from other people: *“I find with this illness, most people are like me, they’ll hide from the public because they’re worried about what people will say. But when you meet likeminded people ….it can do a lot of good.”*

#### Juggling a complexity of health issues

Participants described having to navigate a multitude of health challenges. Most were living with more than one mental illness; five participants additionally managed chronic physical conditions, and five participants spoke about struggling with drug and alcohol addiction. Participants described being overwhelmed by the medication regime required to treat both mind and body. They reported that mental health facilities did not consider or address their physical conditions, and physical health services didn’t consider their mental health needs. This siloed approach hindered their recovery. Ann noted, *“My biggest problem with actively seeking out help… at [mental health facility] is they don’t look after my physical health there because it’s not an acute hospital.”*

Stigma associated with mental ill-health also influenced the treatment of physical conditions. A number of participants described barriers to accessing pain medication for physical health needs, especially if they were also experiencing drug addiction. Eileen, who has a back injury, commented, *“I went to the hospital for pain…because I couldn’t stand the pain in my spine. They weren’t even interested in looking at my spine; just* [saw me as a] *junkie and that stigma sticks.”* Similarly Lara, who had cancer, found, *“they [doctors] wouldn’t give me pain meds, they’d treat me like a drug addict.”* However, some participants, including Lara talk triumphantly of successfully managing the double whammy of physical and mental health issues: *“I’ve done all that on my own and I’ve come out the other side…. If I can do this, I can do anything”*.

## Discussion

While challenges of experiencing mental illnesses, particularly those with illnesses labelled severe and persistent, are well reported, an account of these challenges are absent from much of the recovery-focused literature. Given that recovery and recovery-focused practice are increasingly prominent in policies and mental health service plans internationally [e.g., 8, 9, 10], it is important to ensure that this ‘paradigm’ is inclusive of and relevant to those at an earlier and more complex point of their recovery journey, and does not un-intentionally exclude them. While the recovery experiences of people in the later stages of recovery have been documented (e.g., [17, 28]), there is a need to better understand the experiences of those who are beginning their recovery. Our research findings facilitate the ‘expansion’ of our understanding and framing of recovery by focusing on early recovery. This knowledge is important because insights into consumers’ perspectives and needs early on in their recovery journey can ensure that support services can be more effectively tailored to meet earlier recovery needs and that both consumers, and those that support them, do not feel discouraged by comparing their recovery experiences to accounts of later stage recovery.

A sense of resilience came through these interviews, of strength amidst adversity and a sense of triumph. In all of the interviews people spoke about their hopes for the future and the steps that they had taken towards recovery. However it was not an easy recovery journey for these participants and there was a sense of vulnerability as they recounted past difficulties and ongoing struggles. The results reveal seven key aspects of early recovery: (1) engaging with the challenge of recovery; (2) struggling to find safety and security; (3) grieving for what was and what could have been; (4) seeking and finding hope and purpose; (5) navigating complex relationships; (6) connecting with formal and informal support; and, (7) juggling a complexity of health issues. These findings align with and extend the CHIME (Connectedness, Hope and optimism, Identity, Meaning, Empowerment) model or framework of recovery [15] by adding a layer of early recovery complexity.

Within the CHIME framework ‘connectedness’ refers to positive relationships with peers, family, friends and health professionals. Early in recovery, connectedness is central and our results showed that positive human supports were the greatest facilitator of early recovery and this extended to workers. Helpful workers, were those that valued people as individuals, showed kindness and saw them as experts in their own recovery. These accounts emphasised the importance of personal, human connections, a relational approach in which people are individually valued and their agency recognised despite the perceived ‘messiness’ of their lives at this early stage of recovery. However there are also numerous challenges to overcome for connection. Participants were navigating and learning to ‘leave’ un-helpful relationships behind. They were struggling with loss of relationships and to keep friend and family relationships distinct from carer roles. They were also learning to stand up against health professional practices, and directives that they did not agree with and to seek second opinions.

A second component of the CHIME model is hope and optimism – a belief that a better life is both possible and attainable. For participants in this study, hope and optimism waxed and waned. What was important was repeatedly re-finding them and fighting the urge to withdraw or give up. Identity and regaining a positive sense of self is the third component of CHIME. Participants in this study described battling with self-stigma and seeing beyond the immediate. They described learning to be kinder to themselves but talked little about longer-term visions for their future. Meaning as defined within the CHIME model was also reflected in participant stories. Participants found meaning through doing things for themselves, doing things for others and for some, through religious faith. Parenting roles provided great meaning for many participants. Importantly, meaning and identity were tempered by grieving for the losses associated with having a mental illness, be that loss of employment, loss of connections and family, or loss of previous life dream or aspirations. Finally, empowerment in the CHIME model refers to control over an individual’s life and the decisions about their treatment and supports. Participants were learning and still struggling to assert their views and wishes within the treatment and service context. Some workers facilitated empowerment while others did not. Control over life more broadly was still not in the grasp of most participants. This leads to the aspect of this study that is not captured within the CHIME model: a secure and stable footing. Central to participants’ ability to engage in their recovery was material, interpersonal and environmental stability and security.

Stability of material needs provided a basis from which recovery could be prioritised [7]. The struggle for basic physical needs featured prominently in these interviews. Participants spoke about the need for money, food and above all stable accommodation. The lack of stable, affordable and safe accommodation impacted on consumers’ decision making capacity and their ability to direct their energies towards recovery. This issue of stable accommodation is not new [29] with its historical roots in de-institutionalisation but compounded in recent years by staggering decreases in housing affordability, particularly in Sydney, where this study took place. Housing prices and rent costs have increased up to 70% in Sydney since 2012. The stock or amount of low cost and social housing has not kept up with population increases. Income supports payments have not kept up with rental price increases. Collectively this has resulted in over 40% of Australians on welfare support experiencing housing-related financial stress [30]. There is a need to prioritise these non-clinical aspects that impact upon recovery. Mental health support programs, need to focus holistically on an individual and ensure that these basic needs such as housing security are met. Stability of support from services, families, friends, peers and carers was important to participants. Families of origin were a source of both hope and difficulty. Children provided a purpose and support, but participants struggled with trying to separate out family and child relationships with carer roles. They were also concerned about the impact of their mental illness on others including their children. This period of recovery can cause significant stress for children and other family members, who need to be supported themselves. Reacting to the one-sidedness of current parenting support programs which focus only on the parent living with mental illness, authors recommend the development of ‘family-focused’ programs which support both parent and child to ensure a consistent, individualised and collaborative approach to recovery [31]. The predominance of participant focus upon relationships with others, and the positive and negative impacts of these speak directly to the ‘relational’ approach to recovery recently promoted [32, 33] and calls for extension to the CHIME component of connectedness. A relational approach warns against focusing too heavily on an individual’s inner recovery struggle and neglecting the interpersonal aspects of recovery. Instead programs should focus both on the individual and those relationships closest to them, including their families of origin and particularly their children [32].

### Limitations

As is the case with all qualitative research, the broader relevance of these findings needs to be considered with reference to characteristics of participants. Participants were all engaged in an Australian Sydney-based PIR program. Voluntary participation also means that experiences of early recovery reported here do not reflect experiences of those likely to be at a similarly early point in their recovery journey who chose not to engage in the study. Participants only received invitations to participate if they were deemed by Support Facilitators to be able to participate without undue distress. Results should be considered in the light of this limitation.

## Conclusions

Findings of this study provide further understanding of the early recovery experiences for people living with severe, persistent mental illness and complex needs that align well to, but expand upon the components of the CHIME model. This suggests that a broader conceptualisation is needed within optimistic and aspirational point-of-recovery frameworks if they are to be inclusive of, and relevant to people with severe and persistent mental illness, at an early point of recovery. Recovery at this early stage is not all positive and people described multiple barriers and struggles which impeded their recovery journey. Rather than focusing upon symptoms of illness, participants emphasised relational enablers and barriers. People, and material resources, created physical and emotional safety and support that allowed them to prioritise recovery.

## Abbreviation

PIR: Partners in Recovery
